# Holding of bovine blastocysts at suprazero temperatures using small molecules

**DOI:** 10.1038/s41598-017-10014-9

**Published:** 2017-08-25

**Authors:** Daehwan Kim, Hyeonseok Sul, Yeon-Gil Jung, Sangho Roh

**Affiliations:** 1 Cellular Reprogramming and Embryo Biotechnology Laboratory, Dental Research Institute, BK21, Seoul National University School of Dentistry, Seoul, Republic of Korea; 2 ET Biotech Co. Ltd., Jangsu, Republic of Korea

## Abstract

Although assisted reproductive technology (ART) currently exists, the only embryo preservation technology that is available is cryopreservation. In the present study, small molecules were used to hold embryos at room temperature. The basic medium for embryo holding for a short period of time at 4 °C, 10 °C and 20 °C consisted of 1% BSA non-cryopreservation medium (BNC) instead of fetal bovine serum. To maintain survival and prevent damage during embryo incubation, three candidate small molecules were selected—CHIR99021, Y-27632 and Thiazovivin—and their concentrations were optimized. The viability and hatching rate of embryos incubated at 10 °C were greater for Y-27632-BNC and CHIR99021+Y-27632-BNC compared to BNC. However, the rate was lower for Thiazovivin-BNC compared to BNC. Although there were no surviving embryos after incubation at 20 °C, the viability and hatching rate of embryos significantly increased in Y-27632-BNC and CHIR99021+Y-27632-BNC compared to BNC. The pregnancy rate of embryos incubated at 20 °C was also greater in the CHIR99021+Y-27632-BNC group compared to that in the frozen group. The mechanism by which small molecules enhance survival of embryos during incubation was investigated, and expression of heat shock protein 70 was observed to increase. The findings of this work may be useful in improving ART in the agricultural field.

## Introduction

Cattle are one of the most important livestock in the world. Their reproduction constitutes a large part of the agricultural economy in many countries including milk and meat. Considering this point, it is essential to use and develop assisted reproductive technology (ART) such as artificial insemination, embryo transfer (ET) and embryo production by *in vitro* fertilization (IVF) in this species^1^. However, most ART has focused on improving embryo production, genetic selection and pregnancy rates, while embryo storage has not received as much attention. In numerous instances during ART, embryos require storage for short or long periods of time. Cryopreservation with liquid nitrogen (LN_2_) is generally used for long- as well as short-term embryo storage. However, physical damage is caused during the freeze-thaw process and the damage can limit embryo survival^2^. Thus, only high degree (high quality) embryos that can endure physical damage are normally used, while the rest, even though they still have the potential for full development, are mostly discarded. Moreover, freezing-induced damage reduces pregnancy rates either directly or indirectly^3^. Therefore, it is necessary to improve storage systems to improve ART efficiency.

It has been reported that many cells, including embryos, can be stored at hypothermic temperatures^4, 5^. Recently, it was also documented that bovine embryos could be incubated for 7 days using a simple medium including FBS and that embryos incubated in the medium were able to develop into normal calves^6^. However, the low-temperature embryo holding protocol still had limitations including temperature tolerance, preservative quality, mobility and portability. In addition, it was hard to perform the low-temperature protocol in many farms and sheds, especially on a small scale. So, in order to improve this promising technique, optimization of the holding medium that protects embryos at a wide temperature range including room temperature is needed.

Small molecules are used to maintain the self-renewal and pluripotency of stem cells by inhibiting differentiation^7, 8^. According to these strategies, the inhibition of GSK3 signals is able to activate β-catenin and the results maintain the pluripotent potential for stem cells by suppression of differentiation. Furthermore, the Rho-associated protein kinase (ROCK) pathway increases adhesive molecules and cell survival rates. Recent studies have reported that ROCK inhibitors, specifically Thiazovivin and Y-27632, can effectively maintain the pluripotency and increase the viability of stem cells after freezing and thawing^9, 10^. In cows, several studies have shown that these small molecules are also able to establish embryo-derived stem cells^11–13^. However, little is known about the relationship between small molecules and the resistance to heat stress during short-term embryo holding at a wide temperature range. In addition, no complete mechanism explaining the effects of small molecules on embryo incubation has ever been reported.

In the present study, it was demonstrated that small molecules were able to enhance the survival and hatching rates of embryos after incubation at 20 °C. Moreover, it was shown that the heat shock protein (HSP) family was strongly connected to the mechanism by which small molecules enhanced suprazero temperature incubation.

## Results

### Optimization of the BSA-based medium

To optimize the concentration of BSA for embryo holding without the freezing procedure, called ‘non-cryopreservation,’ the embryos were held for 72 h at 4 °C in holding medium supplemented with BSA at two different concentrations, 1% or 5%. The rates of viability and hatching of the embryos were not significantly different between the 1% and 5% BSA groups (Table S1). Thus, 1% BSA was selected as a suitable concentration and used moving forward as the BSA non-cryopreservation medium (BNC). To confirm system properties, embryos were incubated for 168 h at 4 °C in BNC (Table S2). The viability and hatching rates of the embryos were comparable to that reported in the previous literature^6^. From 24 to 120 h, results showed that the rates of viability and hatching for the embryos incubated in BNC were higher than previous results for those incubated with FBS^6^, implying that BSA may be better suited for non-cryopreservation of bovine embryos.

### Small molecule optimization

To determine whether incubation in BNC was enough to protect embryos from the high temperature at 20 °C, embryos were incubated for 96 h at 20 °C in BNC. However, different from the results at 4 °C, the rate of viability decreased and no embryo hatched during embryo holding for 96 h at 20 °C (Table 1).

**Table 1:** The viability and hatching rates for blastocysts incubated at 20 °C for 96 h.

Time (h)	No. of		
	Blastocysts	Viable embryos (%^†^)	Hatching embryos (%^†^)
24	13	13 (100.0 ± 0.00)	13 (100.0 ± 0.00)
48	14	9 (65.0 ± 5.00)^*^	8 (56.6 ± 3.33)^*^
72	13	1 (8.33 ± 8.33)^**^	0 (0 ± 0.00)^**^
96	10	0 (0 ± 0.00)^**^	0 (0 ± 0.00)^**^

*Data differs significantly from 24 h group at *P* < 0.05, N = 3.

^**^Data differs significantly from 24 h group at *P* < 0.001, N = 3.

^†^Mean ± SEM.

**Figure 1:**
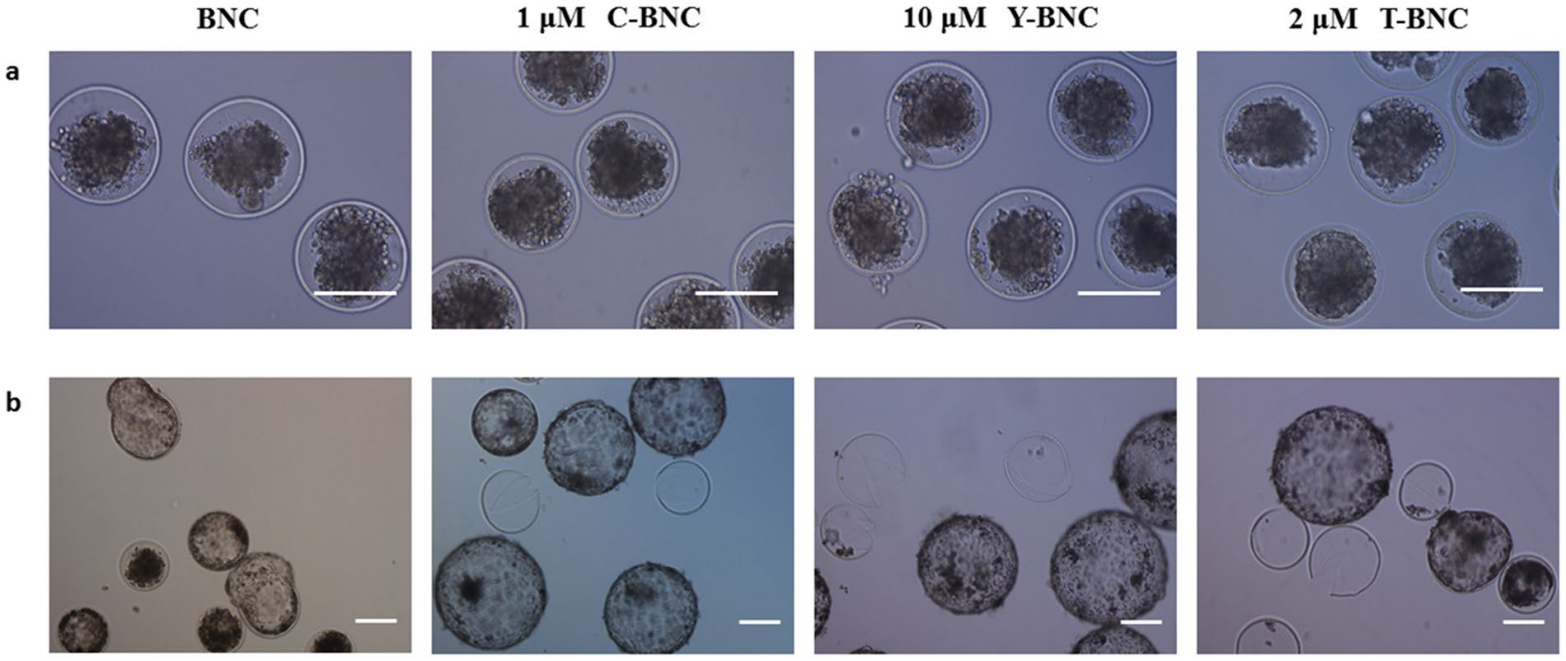
The effects of three small molecules on bovine embryo incubation at 4 °C for 96 h. (**a**) After incubation at 4 °C for 96 h, the morphology of embryos in three different small molecule groups were comparable to those in the BNC group, appearing dark and shrunken. (**b**) After *in vitro* culture for 48 h (recovering period), expanded and hatched embryos were shown in all experimental groups without any morphological differences. Abbreviations are the same as in Table 2. Scale bar = 100 μm.

To improve survival and hatching rates of the embryos after incubation at suprazero temperature, three different small molecules, CHIR99021, Y-27632 and Thiazovivin, were selected. First, the viability and hatching rates of the embryos incubated in BNC with small molecules were evaluated to optimize the concentrations of small molecules (Table 2). According to our data, the optimal concentration of CHIR99021 was determined to be 1 μM, with high survival and hatching rates (92.1 ± 3.95% and 87.9 ± 7.23%, respectively), and 10 μM of Y-27632 showed the highest survival and hatching rates (91.5 ± 4.33% and 78.3 ± 4.13%, respectively). For Thiazovivin, the survival and hatching rates were the highest at 2 μM (83.2 ± 4.20% and 70.5 ± 4.43%, respectively). Although the survival and hatching rates after various combinations of small molecules varied, there were no significant differences in viable embryo morphology (Fig. 1).

**Table 2:** The optimization of small molecule concentration during incubation at 4 °C for 96 h.

		Concentration (μM)	No. of		
			Blastocysts	Viable embryos (%^†^)	Hatching embryos (%^†^)
Single small molecule	BNC	0	14	11 (78.8 ± 1.66)	11 (78.8 ± 1.66)
	C	1	24	22 (92.1 ± 3.95)^*^	21 (87.9 ± 7.23)
		3	24	17 (71.0 ± 2.41)	14 (57.9 ± 4.82)^*^
		6	16	10 (63.3 ± 8.81)	10 (63.3 ± 8.81)
	Y	5	24	19 (79.5 ± 3.21)	18 (75.3 ± 6.81)
		10	23	21 (91.5 ± 4.33)^*^	18 (78.3 ± 4.13)
		20	24	19 (79.5 ± 3.21)	18 (74.7 ± 1.83)
	T	2	24	20 (83.2 ± 4.20)	17 (70.5 ± 4.43)
		10	24	19 (79.5 ± 3.21)	16 (66.8 ± 2.57)
		20	24	19 (80.09 ± 10.89)	16 (67.4 ± 10.32)
Combination of small molecules	C+Y	1 and 10	39	25 (64.7 ± 4.01)	22 (56.1 ± 1.94)
	C+T	1 and 2	39	13 (32.5 ± 8.08)*	10 (24.7 ± 8.53) *

^*^Data differs significantly from BNC at *P* < 0.05, N = 3.

^†^Mean ± SEM.

^‡^Abbreviations: BNC, BSA non-cryopreservation medium; C, CHIR99021; Y, Y-27632; T, Thiazovivin.

### Testing the effect of small molecules on embryo viability at suprazero temperatures

To investigate the effects of small molecules on embryo incubation at suprazero temperatures, embryos were incubated for 96 h at 10 °C in BNC with small molecules: 1 μM CHIR99021-BNC (C-BNC), 10 μM Y-27632-BNC (Y-BNC) or 2 μM Thiazovivin-BNC (T-BNC). Interestingly, the hatching rate was significantly higher in the embryos of Y-BNC group when compared with the embryos of BNC group. In addition, the rates also had a tendency to be greater for those incubated in C-BNC compared to BNC (Table 3). To investigate potential synergistic effects of small molecules, the bovine embryos were incubated in combination groups C+Y-BNC or C+T-BNC for 96 h at 10 °C. The viable and hatching rates of C+Y-BNC group embryos were higher than those of BNC counterpart, while the rates of C+T-BNC group embryos were *vice versa* (Table 3). The viable rate of C+T-BNC group embryos was significantly lower than that of BNC ones.

**Table 3:** The viability and hatching rates for blastocysts incubated in BNC with small molecules at 10 °C for 96 h.

Groups	No. of		
	Blastocysts	Viable embryos (%^**‡**^)	Hatching embryos (%^**‡**^)
BNC^†^	30	18 (60.0 ± 5.77)	11 (36.6 ± 3.33)
C-BNC	34	22 (64.6 ± 1.03)	15 (44.2 ± 1.26)
Y-BNC	34	27 (79.2 ± 3.31)	24 (70.7 ± 5.62)^*^
T-BNC	33	15 (45.4 ± 5.22)	11 (33.3 ± 3.03)
C+Y-BNC	39	25 (63.9 ± 2.80)	22 (56.6 ± 1.66)
C+T-BNC	39	13 (33.3 ± 3.15)^*^	10 (26.1 ± 3.87)

^*^Data differs significantly from BNC at *P* < 0.05, N = 3.

^‡^Mean ± SEM.

^†^Abbreviations are the same as in Table 2.

Different from other small molecules, Thiazovivin reduced survival and hatching rates; embryos incubated in T- and C+T-BNC at 10 °C exhibited lower survival and hatching rates compared to those incubated in BNC.

**Figure 2:**
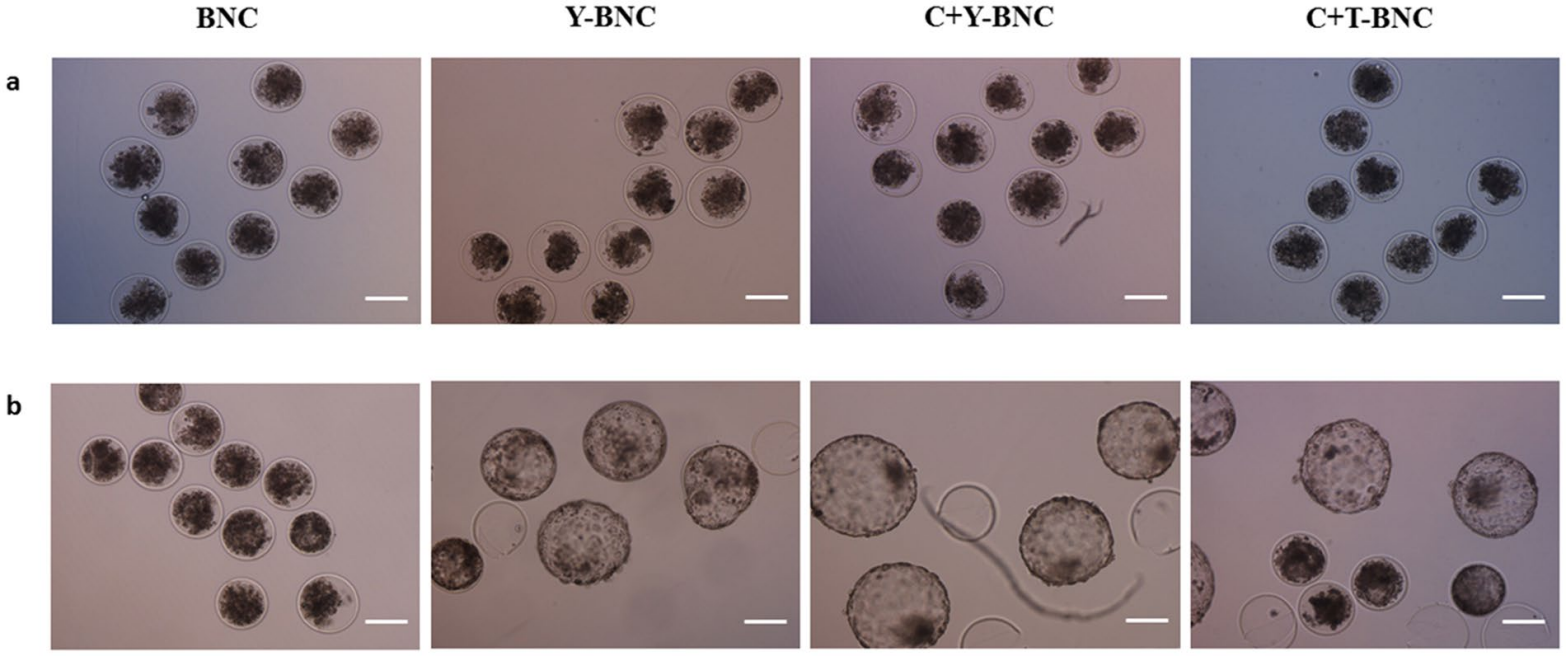
The effects of small molecules on bovine embryo incubation at 20 °C for 72 h. (**a**) After incubation at 20 °C for 72 h, the morphology of the embryos incubated in three different small molecule groups was comparable to those incubated in BNC, appearing dark and shrunken. (**b**) After *in vitro* culture for 48 h (recovering period), expanded and hatched embryos were shown in Y−, C+Y− and C+T-BNC media with no morphological differences. However, there were no living embryos after incubation in BNC. Abbreviations are the same as in Table 2. Scale bar = 100 μm.

To evaluate the effects of small molecules on embryo holding at 20 °C, three different groups-Y−, C+Y− and C+T-BNC—were investigated. Although some embryos survived after incubation in BNC, only two embryos survived and no embryos hatched (Table 4). In contrast, there was a huge upward trend of viability and hatching rates when embryos were incubated with Y, C+Y or C+T. Specifically, the viability and hatching rates of the embryos incubated in Y- (40.0 ± 5.77% and 30.0 ± 5.77%) and C+Y-BNC (50.0 ± 5.74% and 36.7 ± 6.66%) were significantly greater than the rates of those incubated in BNC (Table 4). The morphology observed under light microscopy showed that the embryos in BNC appeared shrunken, and these did not hatch during the 48 h recovering period after incubation (Fig. 2). On the other hand, some embryos in the small molecule-supplemented groups expanded and hatched.

**Table 4:** The viability and hatching rates for blastocysts incubated in BNC with small molecules at 20 °C for 72 h.

Groups	No. of		
	Blastocysts	Viable embryos (%^**‡**^)	Hatching embryos (%^**‡**^)
BNC^†^	30	2 (6.6 ± 6.66)	0 (0.0 ± 0.00)
Y-BNC	30	12 (40.0 ± 5.77)^*^	9 (30.0 ± 5.77)^*^
C+Y-BNC	30	15 (50.0 ± 5.74)^*^	11 (36.7 ± 6.66)^*^
C+T-BNC	30	8 (26.6 ± 3.33)	5 (16.6 ± 8.81)

^*^Data differs significantly from BNC at *P* < 0.05, N = 3.

^‡^Mean ± SEM.

^†^Abbreviations are the same as in Table 2.

### Small molecules enhance the expression of heat shock proteins to improve resistance to suprazero temperature

**Figure 3:**
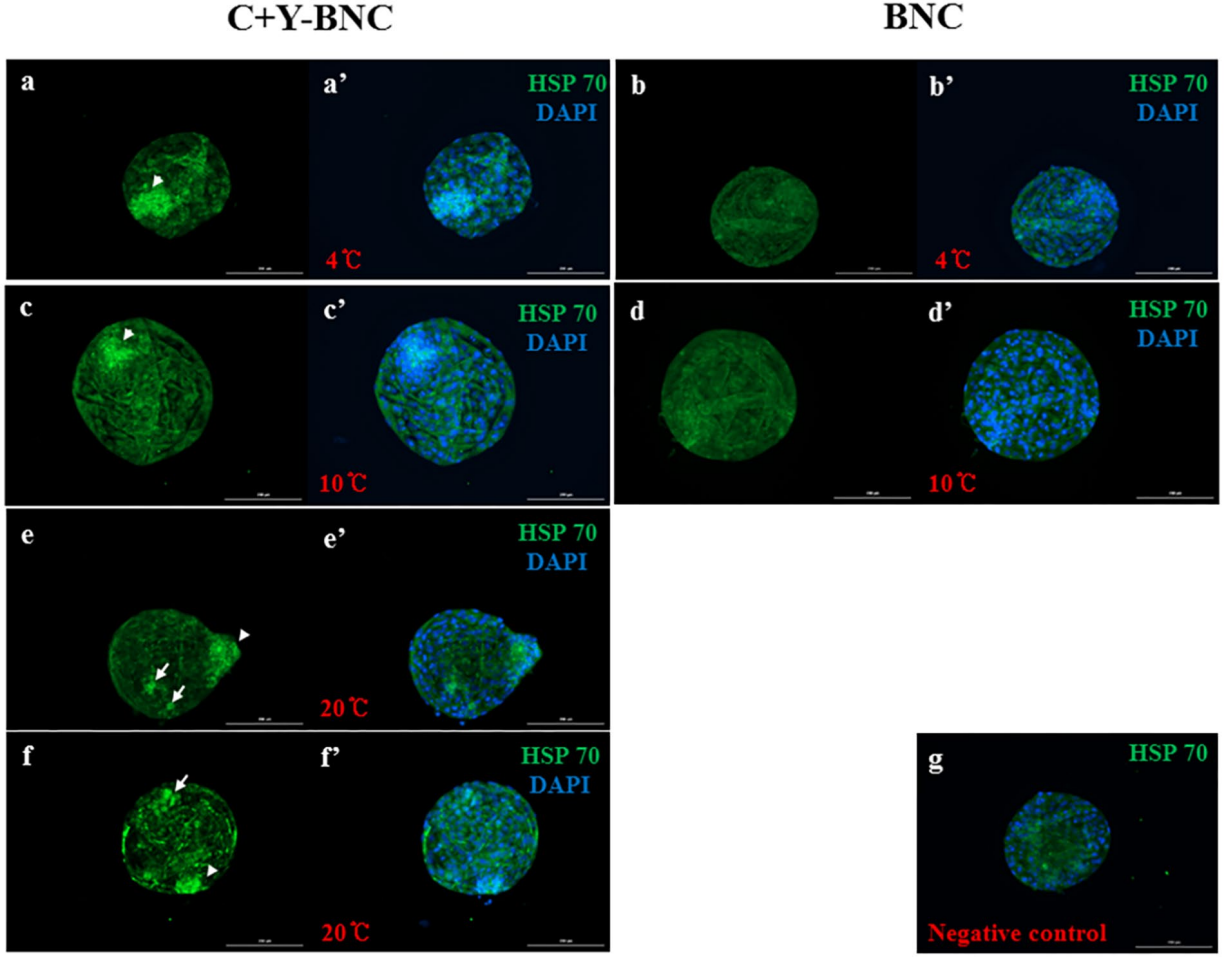
The expression of heat shock protein 70 (HSP70) after incubation with small molecules at 4, 10 and 20 °C. HSP70 expression (green) was observed in embryos incubated in C+Y-BNC at 4, 10 and 20 °C (**a,a′, c,c′, e,e′, f** and **f′**), but not BNC (**b,b′, d**, and **d**′). The arrows point to elevated expression of HSP70 in trophoblasts and the arrow heads point to elevated HSP70 expression in the inner cell mass. The nucleus was stained with DAPI (blue). The negative control was a stained embryo without primary anti-body (**g**). Abbreviations are the same as in Table 2. Scale bar = 200 μm.

To determine the mechanism that promoted suprazero temperature tolerance, HSP60, HSP70 and HSP90 were investigated among embryos incubated in C+Y-BNC. This medium was selected because it dramatically improved the viability and hatching rates of the embryos compared with those incubated in BNC. The expression of HSP70 was greater in embryos grown in C+Y-BNC at 4, 10 and 20 °C compared with those grown in BNC (Fig. 3). Interestingly, HSP70 was dominantly expressed in the inner cell mass (ICM) of the embryos after incubation in C+Y-BNC. However, there were no significant differences after immunostaining analyses for HSP60 and HSP90 (Fig. S1).

### Investigation of pregnancy rates

To confirm if embryos held at 20 °C for 72 h still had the ability to fully develop into normal calves, pregnancy rates after ET were compared among IVP, Frozen, and C+Y-BNC group embryos. Although there was no significant (*P* = 0.055), the pregnancy rate of C+Y-BNC group embryos (48.0 ± 1.38%) tended to be higher than the rate of Frozen group ones (35.8 ± 1.34%) (Table 5). Interestingly, the elevated pregnancy rate for embryos incubated in C+Y-BNC was comparable to that for the IVP group (60.7 ± 1.64; Table 5).

**Table 5:** The comparison of pregnancy rates from the blastocysts of three different origins.

Groups	No. of	
	Blastocysts transferred	Pregnant cows at day 60 (%^‡^)
IVP^*^	186	113 (60.7 ± 1.64)^a^
Frozen^†^	120	43 (35.8 ± 1.34)^b^
C+Y-BNC^††^	127	61 (48.0 ± 1.38)^b^

^a-b^Values with different letters are significantly different (*P* < 0.05).

^*^ *In vitro* produced (IVP) Day 7 blastocysts were directly transferred into the recipients.

^†^Thawed embryos after 72 h of freezing and preserved in liquid nitrogen were transferred into the recipients.

^††^After incubation with small molecules at 20 °C for 72 h, the embryos were transfer into the recipients.

^‡^Mean ± SEM.

^§^Abbreviations are the same as in Table 2.

## Discussion

In this report, we demonstrated that the small molecules CHIR99021, Y-27632 and Thiazovivin permitted short-term incubation for bovine embryos at suprazero temperature by improving survival and hatching rates. Furthermore, it was shown that the mechanism by which these molecules promoted short-term incubation at suprazero temperature involved proteins in the HSP family.

In most domestic animals, the most effective temperature for living and breeding is 10–20 °C. Thus, ET is conducted within this temperature zone to achieve successful reproduction. It is known that the pregnancy rate at dairy farms decreases 5–10% during the hot season, suggesting that bovine embryos may be susceptible to heat stress^14^. However, there have been only a few studies that have investigated the effect of temperature during embryo incubation. Although bovine embryos can be held at 4 °C temporarily^6^, it may not be practical to store and ship it because a special device is needed to maintain the temperature consistently. The method may be difficult to use in a place where there is no electricity, particularly when waiting for optimal conditions for embryo transfer in the farm. Thus, it is necessary to optimize incubation methods in order to permit embryos to survive at a wide temperature range with non-cryopreservation techniques.

FBS-based medium was broadly used for cryopreservation as well as low-temperature incubation^6, 15, 16^. Recently, due to unknown factors, FBS-based media were replaced with purified BSA^17^. In order to avoid interruptions in small molecule pathways by unknown factors, embryo holding at suprazero temperature was conducted with BSA. According to our data, there was no difference between 1% and 5% BSA-based media. Thus, 1% BSA was determined to be a suitable basal medium for non-cryopreservation in cows. Interestingly, survival and hatching rates were analogous to those observed in previous low-temperature incubation studies with FBS, implying that BSA-based medium may be able to replace FBS-based medium for incubation^6^. In this study, to characterize the potential of a non-cryopreservation incubation method at high temperature, embryos were first incubated in BSA-based medium (BNC) at 20 °C for 96 h, but these embryos were not viable. Thus, additional supplements were necessary to support embryo survival during incubation at 20 °C.

To develop a suitable medium for incubation at high temperatures, small molecules related to pluripotency and cell survival were selected. Because pluripotent stem cells are also located in the ICM of embryos, this suggested that the small molecules might be helpful for non-cryopreservation of embryos at high temperatures. In order to apply small molecules to embryo incubation, the optimal concentration of each was determined. Our results showed 1 μM CHIR99021, 10 μM Y-27632 and 2 μM Thiazovivin were optimal concentrations. To investigate the effects of these small molecules on survival and hatching rates, embryos were incubated at 10 °C for 96 h. Survival and hatching rates displayed a tendency to increase in all groups compared with those in the BNC group. Survival and hatching rates tended to elevate in C-BNC and C+Y-BNC group embryos when compared with those in BNC counterpart. In particular, the hatching rate of Y-BNC group embryos was significantly higher than that of BNC ones. These results suggest that CHIR99021 and Y-27632 helped embryos survive and hatch after incubation at 10 °C. On the other hand, survival and hatching rates decreased insignificantly in T- and C+T-BNC media, implying that Thiazovivin may be inappropriate for embryo non-cryopreservation at 10 °C, despite the fact that both Thiazovivin and Y-27632 share signaling targets that are related to cell survival.

Based on results from the experiment at 10 °C, three different groups were selected for the incubation test at 20 °C. However, no embryos survived 96 h of incubation at 20 °C, even those incubated with small molecules. Thus, we investigated if small molecules were able to support embryo incubation at 20 °C for 72 h. Unexpectedly, all media supported embryo survival and most of the embryos hatched. In particular, compared with those in the BNC group, survival and hatching rates significantly increased in Y- and C+Y-BNC, consistent with that of the experiments at 10 °C. Together, our results demonstrated that CHIR99021 and Y-27632, which have cellular targets that are related to pluripotency and cell survival, were able to improve survival and hatching rates and improve non-cryopreservation of bovine embryos at 10 °C and 20 °C for a short period of time.

There was no morphological difference among the experimental groups. All blastocysts showed shrunken morphologies after incubation. The cause of the shrinkage in this study is not clear. However, one of the main reasons may be an unsuitable thermal environment for the embryos (in the body, bovine embryos are staying at 38.5 °C). Shrinkage of blastocysts increases survival and pregnancy rate after cryopreservation^18, 19^. Inappropriate temperature-induced shrinkage of the blastocyst may protect the embryo and have a positive effect on its survival. This is the first reported application of small molecules to hold bovine embryos at suprazero temperature. However, the mechanisms of the molecules responsible for these results still remain elusive.

To clarify these mechanisms, particular attention was paid to the HSP family, which is associated with heat stress. HSP70 promotes cell survival, and HSP90 is essential for cell survival as well as growth^20, 21^. Although it is still controversial as to whether HSP60 supports cell viability, HSP60 is known as a regulator of cell survival^22, 23^. Thus, it was hypothesized that HSP60, HSP70 and/or HSP90 may be involved in the ability of small molecules to improve the survival and hatching rate of embryos after incubation at suprazero temperature. Indeed, expression of HSP70 increased in embryos incubated in C+Y-BNC, compared with those incubated in BNC, and these results were commonly observed after incubation at 4, 10 and 20 °C (Fig. 3). On the other hand, HSP60 and HSP90 expression were not observed in embryos grown in C+Y-BNC, implying that these molecules were not included in the mechanism against suprazero temperature tolerance. Together, these data suggested that CHIR99021 and Y-27632 may have induced HSP70, but not HSP60 or HSP90, and facilitated incubation of embryos at 20 °C for a short period of time.

Although elevated HSP70 expression was observed in whole embryos, it was dominantly expressed in ICM. This may be because cells in embryos are densely located in the ICM. Additionally, this might also be due to the effects of CHIR99021. Previous studies have shown that CHIR99021 is able to support the pluripotency of stem cells^8, 24^, whereas it is not essential for the proliferation of trophoblast stem cells^25^. Considering this literature, HSP70 might be dominantly expressed in ICM via induction by CHIR99021.

The pregnancy rate for embryos incubated with small molecules was then tested. The pregnancy rate of C+Y-BNC group embryos tended to be higher than cryopreservation group, although there was no significance (*P* = 0.055). Moreover, the rate was comparable to embryos produced *in vitro* without any preservation, implying that embryos incubated in C+Y-BNC functioned similar to those that were not stored. This study suggests that BNC with small molecules may be appropriate for short-term incubation of the embryos. This system, which allows embryo’s greater stability against heat stress, might better allow for flexibility in responding to the estrous cycle of surrogate cows. This, in turn, could enhance the efficiency of ART for increased production.

In conclusion, we observed that two small molecules that influence pluripotency and cell viability, CHIR99021 and Y-27632, were able to permit embryos to survive at a broad temperature range (4 to 20 °C), and demonstrated that the mechanism was strongly connected to HSP70 expression. Our suprazero temperature holding system can be used to avoid damages from cryopreservation which enables to deliver the embryos to dairy farms with low cost. This approach may be useful for distribution of cattle genetics without the need for LN_2_ temperatures and enhance livestock industry, for example, holding the embryos in ice water-contained thermos may allow the embryos to be transported by train or airplane for several hours without any electronic devices and/or LN_2_.

## Materials and Methods

### Chemicals

Inorganic and organic compounds were purchased from Sigma-Aldrich Korea (Yong-in, Korea) and all liquid medium and supplements were purchased from Life Technologies (Grand Island, NY, USA) unless otherwise indicated.

### Oocyte recovery and *in vitro* maturation (IVM)

Immature Cumulus-oocyte complexes (COCs) were retrieved by ovum pick-up (OPU). The modified OPU was performed as previously described^26^. Briefly, each visible follicle (≥2 mm in diameter) was aspirated using Ibex® EVO^TM^ (E.I. Medical Imaging, Loveland, CO, USA), a disposable 18 gauge × 90 mm hypodermic needle (Jeil tech Co., Seoul, Korea) connected to a 50 ml conical tube (Corning Life Sciences, Lowell, MA, USA), and a vacuum pump (Gast Manufacturing, Benton Harbor, MI, USA) with a negative pressure of 10–12 ml of water/min. The maturation step was conducted with IVMD 101, which is serum-free maturation medium (Functional Peptides Research Institute, Higashine, Japan) according to the company’s instructions. Briefly, COCs that were enclosed by more than three layers of compact cumulus cells and an evenly granulated ooplasm were selected for IVM. Selected COCs were cultured in 4-well culture dishes (Nunc, Roskilde, Denmark) containing 500 μl of IVMD 101 under warm, gas-equilibrated mineral oil for 20–22 h at 38.5 °C, 5% CO_2_.

### *In vitro* production of bovine fertilized embryos

Fertilized embryos were produced with IVF 100, which is serum-free fertilization medium (Functional Peptides Research Institute, Higashine, Japan) according to the company’s instructions. The expanded COCs were washed twice in IVF 100 and placed into 45 μl drops of IVF 100 under mineral oil. Frozen semen straws from the HanWoo were thawed in a 37 °C water bath and transferred to a 15 ml centrifuge tube with 4 ml IVF 100, and centrifuged at 600 *g* for 10 min. The supernatant was removed, re-suspended in 4 ml IVF 100 and centrifuged at 600 *g* for 10 min. After removal of the supernatant, 5 μl of the sperm suspension (1 × 10^7^ cells/ml) was introduced to the IVP drop, resulting in a final sperm concentration of 1 × 10^6^ cells/ml. Incubations were carried out at 39 °C in 5% CO_2_ for 6 h. Then, the embryos were washed 3 times with IVMD 101 and cultured in an IVMD 101 drop for 24 h. Then, denuded fertilized oocytes were transferred to 100 μl of IVD 101, which is serum-free development medium (Functional Peptides Research Institute, Higashine, Japan), for 7 days at 38.5 °C in a humidified gas environment of 5% CO_2_, 5% O_2_ and 90% N_2_. The culture drops were covered in mineral oil and 10 to 15 embryos were placed in each drop.

### Incubation of bovine embryos at 4 °C, 10 °C, and 20 °C

Bovine embryos were washed three times in each test medium and loaded in a 0.25 ml plastic straw (FHK, Tokyo, Japan), as illustrated in Fig. S2. The straw was hermetically sealed. Then, the embryos were incubated at 4, 10 or 20 °C for 72 or 168 h.

### The evaluation of viability and hatching rate after culturing at 4, 10 or 20 °C

IVP-derived blastocysts were incubated for 96 h at 4, 10 or 20 °C in test media. Following incubation, the embryos were transferred and washed three times with IVD 101. The embryos were incubated with IVD 101 for 48 h at 38.5 °C under 5% CO_2_, 5% O_2_ and 90% N_2_ in the air with high humidity and were assessed for viability and hatching rate. The viability and hatching rates of the embryos were estimated at 24 and 48 h. Then, embryos that appeared dark and shrunken with no cell proliferation or cellular integrity were judged to have degenerated^6^. Embryos that had made a clear breach of the zona pellucida with the trophectoderm were classified as viable blastocysts. In addition, embryos that exhibited discarded zona pellucida were classified as hatching blastocysts.

### Optimizing the concentrations of bovine serum albumin (BSA)

IVP-derived blastocysts were incubated for 72 h at 4 °C in a basic medium containing medium 199 (CN-11150, Thermo Scientific, Logan, UT, USA), 25 mM HEPES and 1% or 5% BSA. The loaded straws were placed in incubators at 4, 10 or 20 °C. Then, the incubation and evaluation of bovine embryos were assessed as above.

### Treatment with small molecules

IVP-derived blastocysts were incubated for 72 or 96 h at 4, 10 or 20 °C in 1% BSA incubation medium plus various combination of three small molecules: CHIR99021 (S-2924; Selleck Chemicals, Breda, the Netherlands), Y-27632 (S-1049; Selleck Chemicals) or Thiazovivin (S-1459; Selleck Chemicals). The small molecules were investigated for incubation at different concentrations (CHIR99021: 1, 3 and 6 μM; Y-27632: 5, 10 and 20 μM; Thiazovivin: 2, 10 and 20 μM). Then, the incubation and evaluation of bovine embryos were assessed as described above.

### Immunofluorescence staining

Immunofluorescence staining was performed according to a standard protocol^27^. Bovine blastocysts were fixed in 4% paraformaldehyde, permeabilized with 0.25% Triton X-100 and blocked with 1% BSA in phosphate-buffered saline (PBS). The fixed cells were immunostained with antibodies against HSP60 (sc-59567; Santa Cruz Biotechnology, Inc., Santa Cruz, CA, USA), HSP70 (sc-66048; Santa Cruz Biotechnology) and HSP90 (sc-7947; Santa Cruz Biotechnology), followed by incubation with secondary antibodies FITC-conjugated goat anti-mouse IgG (A11001, Life Technologies) and FITC-conjugated anti-rabbit IgG (AP132F, Millipore). The treated cells were covered with slow-fade anti-fade with DAPI (SlowFadeGold^TM^ with DAPI, Life Technologies) for nuclear staining and covered with a glass coverslip. Images were captured with a fluorescence microscope (DM5000B, Leica, Bensheim, Germany).

### Evaluation of pregnancy rates

All animals received humane care and all experiments with live animals were performed by a licensed veterinarian in accordance with the Korean Guidelines of Livestock Industry Act (article no. 11 and clause no. 1).

HanWoo embryos (morula to blastocyst stages) were produced *in vitro*. They were washed three times in medium 199 supplemented with 1% BSA and 25 mM HEPES and transferred into medium 199 supplemented with 1% BSA, 25 mM HEPES, 1 μM CHIR99021 and 10 μM Y-27632. Then, the mixture with embryos was loaded into the straw as shown in Fig. S2. The loaded straws were placed in an incubator set to 20 °C. After 72 h, the incubated embryos were transferred into IVD 101 and washed three times in the same medium. Suitable embryos were selected under a light microscope and transferred to the recipients (one embryo per recipient). Pregnancy was determined by Ibex^®^ EVO^TM^ (E.I. Medical Imaging) on day 60 of gestation.

### Statistical analysis

All values are expressed as mean ± SEM. To determine significance between two groups, comparisons were made using Fisher’s exact test by with SPSS Statistics 23.0 software (SPSS, Inc., Chicago, IL, USA). *P* < 0.05 was considered significant.

## Electronic supplementary material

Supplementary Information

## References

[1] Betteridge KJ (2003). A history of farm animal embryo transfer and some associated techniques. Anim Reprod Sci.

[2] Hasler JF, Hurtgen PJ, Jin ZQ, Stokes JE (1997). Survival of IVF-derived bovine embryos frozen in glycerol or ethylene glycol. Theriogenology.

[3] Hasler JF (2001). Factors affecting frozen and fresh embryo transfer pregnancy rates in cattle. Theriogenology.

[4] Chang MC (1947). Normal Development of Fertilized Rabbit Ova Stored at Low Temperature for Several Days. Nature.

[5] Hafez ES (1961). Storage of Rabbit Ova in Gelled Media at 10 Degree C. J Reprod Fertil.

[6] Ideta A (2013). A simple medium enables bovine embryos to be held for seven days at 4 degrees C. Sci Rep.

[7] Chen S (2006). Self-renewal of embryonic stem cells by a small molecule. Proc Natl Acad Sci USA.

[8] Ying QL (2008). The ground state of embryonic stem cell self-renewal. Nature.

[9] Xu Y (2010). Revealing a core signaling regulatory mechanism for pluripotent stem cell survival and self-renewal by small molecules. Proc Natl Acad Sci USA.

[10] Mitalipov S, Wolf D (2009). Totipotency, pluripotency and nuclear reprogramming. Adv Biochem Eng Biotechnol.

[11] Pashaiasl M, Khodadadi K, Holland MK, Verma PJ (2010). The efficient generation of cell lines from bovine parthenotes. Cell Reprogram.

[12] Kim D, Park S, Jung YG, Roh S (2015). *In vitro* culture of stem-like cells derived from somatic cell nuclear transfer bovine embryos of the Korean beef cattle species, HanWoo. Reprod Fertil Dev.

[13] Park S, Kim D, Jung YG, Roh S (2015). Thiazovivin, a Rho kinase inhibitor, improves stemness maintenance of embryo-derived stem-like cells under chemically defined culture conditions in cattle. Anim Reprod Sci.

[14] Burke JM (1996). Evaluation of timed insemination using a gonadotropin-releasing hormone agonist in lactating dairy cows. J Dairy Sci.

[15] Lindner GM, Ellis DE (1985). Refrigeration of Bovine Embryos. Theriogenology.

[16] Wilmut I, Rowson LE (1973). Experiments on the low-temperature preservation of cow embryos. Vet Rec.

[17] Cui XS, Jeong YJ, Lee HY, Cheon SH, Kim NH (2004). Fetal bovine serum influences apoptosis and apoptosis-related gene expression in porcine parthenotes developing *in vitro*. Reproduction.

[18] Vanderzwalmen P (2002). Births after vitrification at morula and blastocyst stages: effect of artificial reduction of the blastocoelic cavity before vitrification. Hum Reprod.

[19] Mukaida T, Oka C, Goto T, Takahashi K (2006). Artificial shrinkage of blastocoeles using either a micro-needle or a laser pulse prior to the cooling steps of vitrification improves survival rate and pregnancy outcome of vitrified human blastocysts. Hum Reprod.

[20] Nylandsted J (2004). Heat shock protein 70 promotes cell survival by inhibiting lysosomal membrane permeabilization. J Exp Med.

[21] Neckers L, Ivy SP (2003). Heat shock protein 90. Curr Opin Oncol.

[22] Samali A, Cai J, Zhivotovsky B, Jones DP, Orrenius S (1999). Presence of a pre-apoptotic complex of pro-caspase-3, Hsp60 and Hsp10 in the mitochondrial fraction of jurkat cells. EMBO J.

[23] Shan Y (2003). Hsp10 and Hsp60 modulate Bcl-2 family and mitochondria apoptosis signaling induced by doxorubicin in cardiac muscle cells. J Mol Cell Cardiol.

[24] Ye, S. D. *et al*. Pleiotropy of Glycogen Synthase Kinase-3 Inhibition by CHIR99021 Promotes Self-Renewal of Embryonic Stem Cells from Refractory Mouse Strains. *PloS one* **7**, 10.1371/journal.pone.0035892 (2012). 10.1371/journal.pone.0035892 PMC3335080 22540008

[25] Ohinata Y, Tsukiyama T (2014). Establishment of trophoblast stem cells under defined culture conditions in mice. PloS one.

[26] Pontes JHF (2011). Ovum pick up, *in vitro* embryo production, and pregnancy rates from a large-scale commercial program using Nelore cattle (Bos indicus) donors. Theriogenology.

[27] Watanabe Y (2004). Conversion of myoblasts to physiologically active neuronal phenotype. Genes Dev.

