# Supplementary material for: Holding of bovine blastocysts at suprazero temperatures using small molecules
Source: Sci Rep. 2017 Aug 25;7:9490. doi: 10.1038/s41598-017-10014-9 (PMC5573397; doi:10.1038/s41598-017-10014-9)
Supplement: Supplementary file 1 — Supplementary Information [file 41598_2017_10014_MOESM1_ESM.pdf]

Supplementary information for

Holding of bovine blastocysts at suprazero temperatures using small molecules

Daehwan Kim[1], Hyeonseok Sul[2], Yeon-Gil Jung[2] and Sangho Roh[1,*]

[1]Cellular Reprogramming and Embryo Biotechnology Laboratory, Dental Research Institute, BK21, Seoul National University School of Dentistry, Seoul, Republic of Korea

[2]ET Biotech Co. Ltd., Jangsu, Republic of Korea

[*]Corresponding author: Sangho Roh, D.V.M., Ph.D.

Professor, Cellular Reprogramming and Embryo Biotechnology Laboratory, Seoul National University School of Dentistry, 1 Gwanak-ro, Gwanak-gu, Seoul 08826, Republic of Korea

E-mail: sangho@snu.ac.kr, Phone: +82 2 880 2333

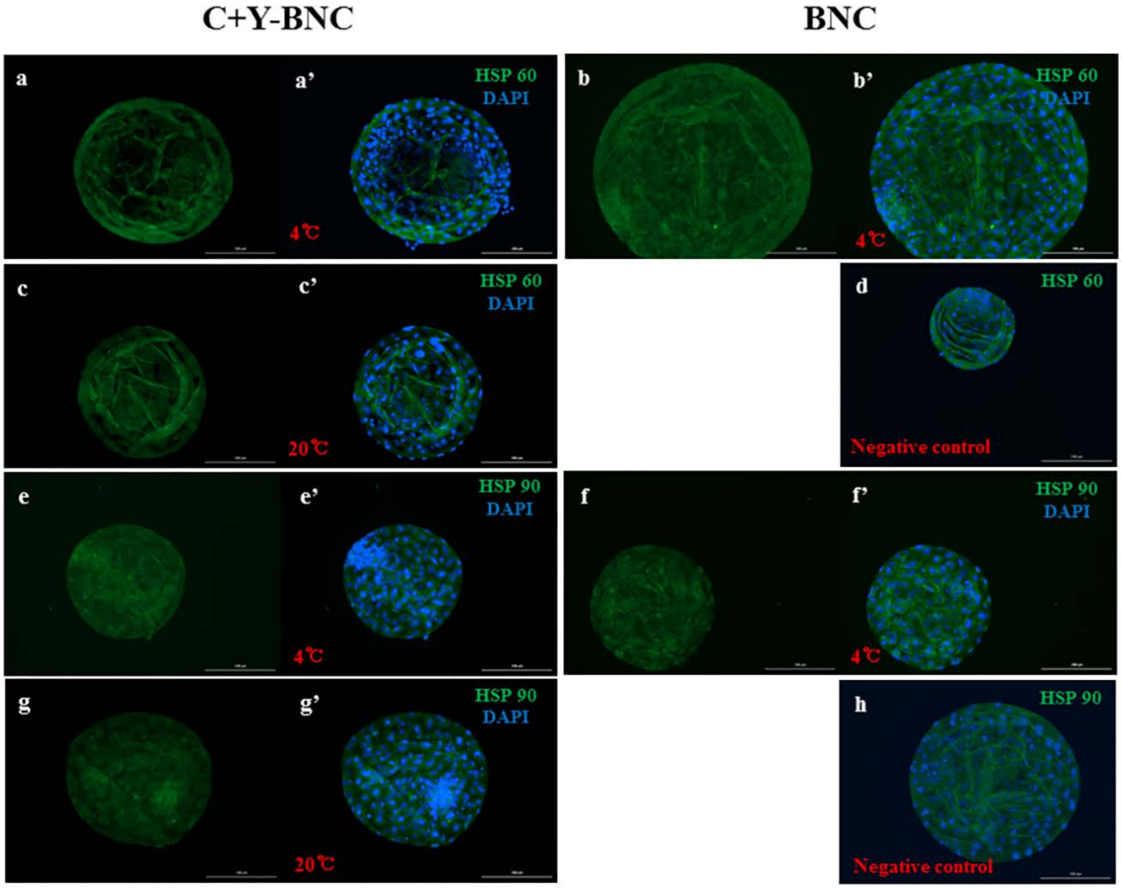

Figure S1. The expression of heat shock protein 60 (HSP60) and heat shock protein 90 (HSP90) after incubation with small molecules at 4 and 20°C. Elevated expression of HSP60 was not detected in embryos incubated at 4°C in BNC or C+Y-BNC (a, a', b and b'). Moreover, embryos incubated at 20°C in C+Y-BNC also did not show elevated expression of HSP60 (c and c'). The expression of HSP90 did not appear to change in embryos incubated at 4°C in either BNC or C+Y-BNC (e, e', f and f'). In addition, embryos incubated at 20°C in C+Y-BNC also did not exhibit changes in expression of HSP90 (g and g'). The nucleus was stained by DAPI (blue). The negative control was a stained embryo without primary anti-bodies (d and h). Abbreviations are the same as in Table 2. Scale bar = 200 μm.

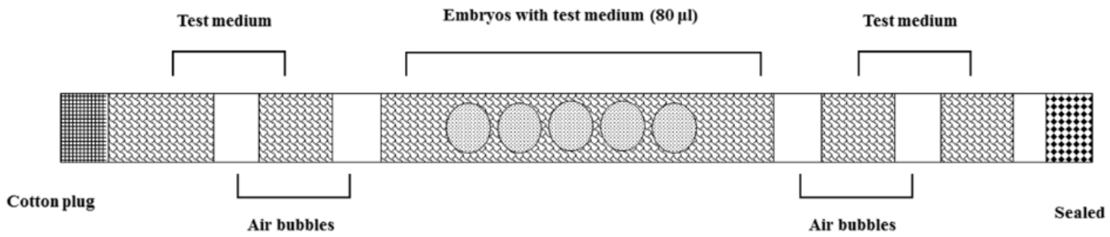

Figure S2. A schematic representation of embryo loading in the straw.

Table S1. BSA concentration optimization in holding medium

Percentage of BSA	No. of		
	Blastocysts	Viable embryos (%[‡])	Hatching embryos (%[‡])
1	46	41 (89.1±2.09)	38 (82.6±2.04)
5	42	38 (90.5±2.00)	35 (82.7±5.47)

[‡] Mean ± SEM

[†]Abbreviations are the same as in Table 2.

Table S2. The viability and hatching rates for blastocysts incubated at 4°C for 168 h

Time (h)	No. of		
	Blastocysts	Viable embryos (%[‡])	Hatching embryos (%[‡])
24	50	50 (100.0±0.00)	49 (98.0±1.96)
48	60	60 (100.0±0.00)	56 (93.3±1.66)
72	100	93 (93.0±0.27)	87 (86.6±1.92)
96	40	36 (90.0±0.00)	32 (78.3±4.41)
120	46	35 (75.9±4.66) [*]	22 (47.9±2.83)
168	22	10 (45.8±4.16) [*]	8 (36.1±1.38)

[*] **Data differ significantly from 24 h group at *P* < 0.05, N=3.**

[‡] **Mean ± SEM**

[†]**Abbreviations are the same as in Table 2.**
